# Assessment of the Microbiota in Microdissected Tissues of Crohn's Disease Patients

**DOI:** 10.1155/2012/505674

**Published:** 2011-12-07

**Authors:** Gert De Hertogh, Bart Lemmens, Peter Verhasselt, Ronald de Hoogt, Xavier Sagaert, Marie Joossens, Gert Van Assche, Paul Rutgeerts, Severine Vermeire, Jeroen Aerssens

**Affiliations:** ^1^Department of Morphology and Molecular Pathology, University Hospitals KULeuven, Minderbroedersstraat 12, 3000 Leuven, Belgium; ^2^Department Research Informatics & Integrated Genomics, Janssen Pharmaceutical Companies of Johnson & Johnson, Turnhoutseweg 30, 2340 Beerse, Belgium; ^3^Department of Gastroenterology, University Hospitals KULeuven, Herestraat 49, 3000 Leuven, Belgium

## Abstract

The microbiota of the gastrointestinal tract is frequently mentioned as one of the key players in the etiopathogenesis of Crohn's disease (CD). Four hypotheses have been suggested: the single, still unknown bacterial pathogen, an abnormal overall composition of the bowel microbiota (“dysbiosis”), an abnormal immunological reaction to an essentially normally composed microbiota, and increased bacterial translocation. We propose that laser capture microdissection of selected microscopic structures, followed by broad-range 16S rRNA gene sequencing, is an excellent method to assess spatiotemporal alterations in the composition of the bowel microbiota in CD. Using this approach, we demonstrated significant changes of the composition, abundance, and location of the gut microbiome in this disease. Some of these abnormal findings persisted even after macroscopic mucosal healing. Further investigations along these lines may lead to a better understanding of the possible involvement of the bowel bacteria in the development of clinical Crohn's disease.

## 1. Introduction

Crohn's disease (CD) is characterized by chronic, segmental, transmural inflammation of the entire gastrointestinal tract. Although the exact etiology of the disease is still unclear, it is generally assumed to originate from immunologic changes induced by environmental influences in a genetically suspectible host. The first discovered CD susceptibility gene was the Caspase Recruitment Domain 15 (*CARD15*) on chromosome 16q [1, 2]. The protein product of this gene is present in monocytes, dentritic cells, Paneth cells, and intestinal epithelial cells. It recognizes a breakdown product of the bacterial cell wall, muramyl dipeptide, via its carboxyterminal leucine rich repeat region (LRR). Subsequently, it activates NF*κ*B. Three single nucleotide polymorphisms (SNPs) of the *CARD15* gene (Arg702Trp-SNP8, Gly908Arg-SNP12 and Leu1007fsinsC-SNP13) are associated with CD in the Caucasian population [3–7]. These 3 SNPs may interfere with the function of the LRR, potentially leading to a disturbance of the normal relationship between the human host and their bowel microbiota. Bacteria have indeed been suggested as one of the most important environmental factors in the pathogenesis of CD. Currently, four hypotheses are proposed. First, the disease may be caused by a single, still unidentified bacterial pathogen (*Mycobacterium avium* subsp. *paratuberculosis* (MAP) and adherent-invasive *Escherichia coli* are possible candidates) [8–11]. Second, the normal balance between beneficial and harmful bacterial species in the mucosa-associated microbiota may be disturbed, so-called “dysbiosis” [12–14]. Third, the mucosa may be abnormally permeable to bacteria or their products (increased bacterial translocation) [12]. Finally, the immune system may react excessively to a normally composed bowel microbiota [15]. If any of these four assumptions are true, bowel biopsies from carriers of the 3 CD-associated *CARD15* SNPs may contain unexpected bacteria or an abnormally composed microbiota in unusual locations.

Testing this hypothesis is complicated by the fact that 70 to 80% of the intestinal bacteria are currently unculturable because of fastidious or even unknown growth requirements. Culture-independent, molecular detection, and identification techniques are therefore recommended [16]. One commonly used approach is based on the structure of the 16S ribosomal RNA (rRNA) gene. This 1550 base pair long gene is found exclusively in bacteria. Its presence on the bacterial chromosome in one or more copies is necessary for normal growth and metabolism. The 16S rDNA contains both highly conserved and hypervariable (V) regions, allowing the construction of universal primers and facilitating bacterial identification up to the (sub)species level, respectively [17]. This technique has been commonly applied to faecal samples and mucosal biopsies [18]. However, a combination with laser-capture microdissection (LCM) would allow to allocate individual bacterial sequences to specific tissue compartments (e.g., the muscular layer of the bowel wall) or even microscopic lesions of interest, such as ulcers, dilated lymph vessels with surrounding inflammatory infiltrate, and granulomas. We have applied this combined approach to surgical biopsies obtained from CD patients, and we have compared our results with those obtained in (non)microdissected tissue samples from disease and healthy controls.

## 2. Materials and Methods

### 2.1. Patients

**Figure 1:**
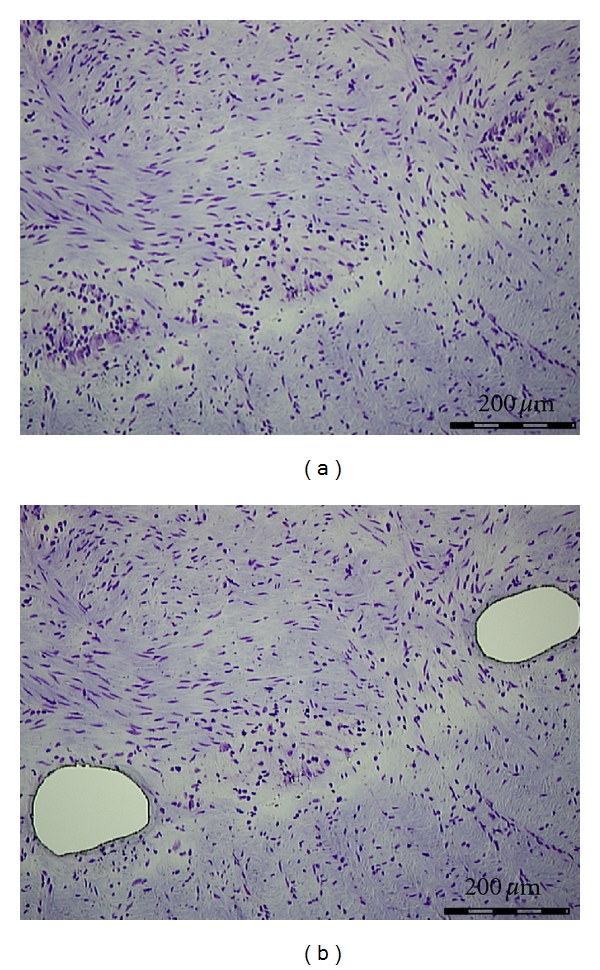
Myenteric plexus before and after microdissection (Cresyl violet, ×50).

Four CD patients, 6 disease controls (acute nonchronic gastrointestinal tract inflammation), and 3 healthy controls (normal mucosa at a large distance from a nonstenosing colorectal adenocarcinoma) were included (Table 1). Genotypes for the 3 CD-associated *CARD15* SNPs were determined for the CD patients and the healthy controls using a salting out procedure starting from venous blood [19]. One to six transmural biopsies were obtained from (ileo)colectomy specimens of these patients after longitudinal opening of the bowel wall and flushing with tap water to remove residual bowel contents. For the disease controls, mucosal biopsies were obtained during colonoscopy. All biopsies were snap-frozen and stored at −80°C. One 5 *μ*m-thick slide from each biopsy was haematoxylin-eosin stained and evaluated microscopically to confirm the diagnosis and, if applicable, to select areas suitable for LCM (Tables 2 and 3, Figure 1).

### 2.2. Laser-Capture Microdissection and DNA Extraction

For LCM, two to four 14 *μ*m-thick tissue sections were prepared using presterilized microtome blades and UV-irradiated PALM MembraneSlides (PALM Microlaser Technologies AG, Bernried, Germany). Sections were fixed for 1 minute in 100% ethanol, dried for 15 min at 37°C, and hydrated in a decreasing concentration range of ethanol. After hydration, the slides were stained with Mayer's haematoxylin for 1 min, dehydrated in an increasing concentration range of ethanol, and air-dried under a fume hood for 5 min. The PALM Robot-MicroBeam System was used for microdissection and dissected tissue was captured in inverted Eppendorf tube caps filled with 50 *μ*L TE buffer (10 mM Tris-HCl pH 8.0, 1 mM EDTA). Endoscopic biopsies and lymph node tissues harvested from surgical specimens were submitted to DNA extraction directly.

Tissue was digested overnight with 50 *μ*L 2x digestion buffer (SDS 2%, 50 mM EDTA, 0.12% proteinase K) at 55°C followed by heat inactivation of proteinase K at 90°C (25 min). DNA was extracted using phenol : chloroform : isoamylacohol (25 : 24 : 1) in Heavy Phase Lock Gel tubes (Eppendorf AG, Hamburg, Germany) and dissolved in 40 *μ*L autoclaved TE buffer for storage at −20°C. D3S3332, a human genomic DNA marker with a length of 215 base pairs (GenBank: Z38904; forward primer: 5′-GCATTTAATGCACTAGATGCTCT-3′; reverse primer: 5′-CTTTAAATGCCAATTACAGTGCA-3′), was amplified from all specimens guaranteeing DNA extraction efficiency and excluding the presence of PCR inhibitors (data not shown).

### 2.3. PCR Amplification of 16S rDNA

A 998 base pair DNA fragment including the V3 to V9 regions of the 16S rRNA gene was amplified from all samples using a two rounds, heminested PCR and universal primers (1st round, primer pair: 342f/1512r; 2nd round: 515f/1512r) (Table 4). For the first round, 4 *μ*L template DNA solution was added to 26 *μ*L PCR mixture, obtaining final concentrations of 1x PCR buffer, 5.5 mM MgCl_2_, 0.2 mM dNTPs, 0.25 *μ*M of each primer and 0.75 U AmpliTaq Gold DNA polymerase (Applied Biosystems, Nieuwerkerk a/d Ijssel, The Netherlands). DNA was first denatured at 95°C for 10 minutes. Then a first PCR round of 30 cycles (denaturation 95°C, 30 sec/annealing 55°C, 30 sec/extension 72°C, 2 min) was performed on a Tetrad-2 Thermal Cycler (Bio-Rad laboratories Inc, Calif, USA). Four *μ*L of unpurified product of this round was used as template for the second PCR round, which also consisted of 30 cycles. Positive and negative controls (4 *μ*L of a 1/100 diluted solution of *Escherichia coli *in LB-medium with an optical density of 0.058, and 4 *μ*L of TE buffer, resp.) were processed simultaneously in every round. All solvents and plastic consumables were sterilized before use.

### 2.4. PCR Product Analysis

The products of the 2nd PCR round were analysed by standard agarose gel electrophoresis. Amplified DNA of the predicted size was excised and purified using the QIAquick Gel Extraction Kit (QIAGEN Benelux BV, Venlo, The Netherlands). Purified PCR products were ligated into pCR 2.1-TOPO plasmid vector using the TOPO TA Cloning Kit. Plasmids were then amplified in TOP10 One Shot Electrocompetent Cells (Invitrogen NV, Merelbeke, Belgium). Transformed cells were cultured, and amplified plasmids obtained from 8 to 48 randomly selected colonies per sample were isolated and purified using the QIAprep Turbo BioRobot Kit on a Biorobot 8000 Workstation (QIAGEN GmbH, Hilden, Germany). 16rDNA sequences were then determined by cycle sequencing using BigDye Terminator (Applied Biosystems Inc, Calif, USA) with M13F (5′-GTAAAACGACGGCCAG-3′) and M13R (5′-CAGGAAACAGCTATGAC-3′) as primers. The sequences were analysed on an ABI PRISM 3730 sequencer (Applied Biosystems) and trimmed to remove vector sequence. All sequences longer than 100 nucleotides and having a greater than 98% similarity were clustered into singular operational taxonomic units (OTUs) using the ClustalW program (version 1.83, European Bioinformatics Institute), whereby OTUs are defined as groups of sequences sharing at least 98% similarity [20]. For every OTU, the sequence with the lowest number of ambiguous nucleotides was analysed with the Basic Local Alignment Search Tool (BLAST 2.2.24 release, National Center for Biotechnology Information) against the GenBank database (release 182.0). Human and vector sequences were excluded from further analysis. Possible chimeras were likewise excluded after detection using the Chimera_Check program (version 2.7) of the Ribosomal Database Project-II (RDP-II, release 9.46). An OTU was identified as a particular species when it showed at least 98% similarity with a cultured, phenotypically identified bacterial species in the databank [21]. Sequences not achieving the 98% limit were assigned to the family level of the taxonomical hierarchy proposed in *Bergey's Manual of Systematic Bacteriology* [22] using the classifier tool of the Ribosomal Database Project-II with a confidence threshold of 80%. To estimate the diversity within each sample, the cumulative number of noncontaminant bacterial families was plotted against the cumulative number of analysed bacterial sequences.

### 2.5. Statistics

Samples were compared for the abundance of various bacterial taxa using the Fisher's Exact test. *P* values <0.05 were considered statistically significant.

## 3. Results

### 3.1. PCR Yield and Comparison of Microdissected and Non-Microdissected Tissues

**Figure 2:**
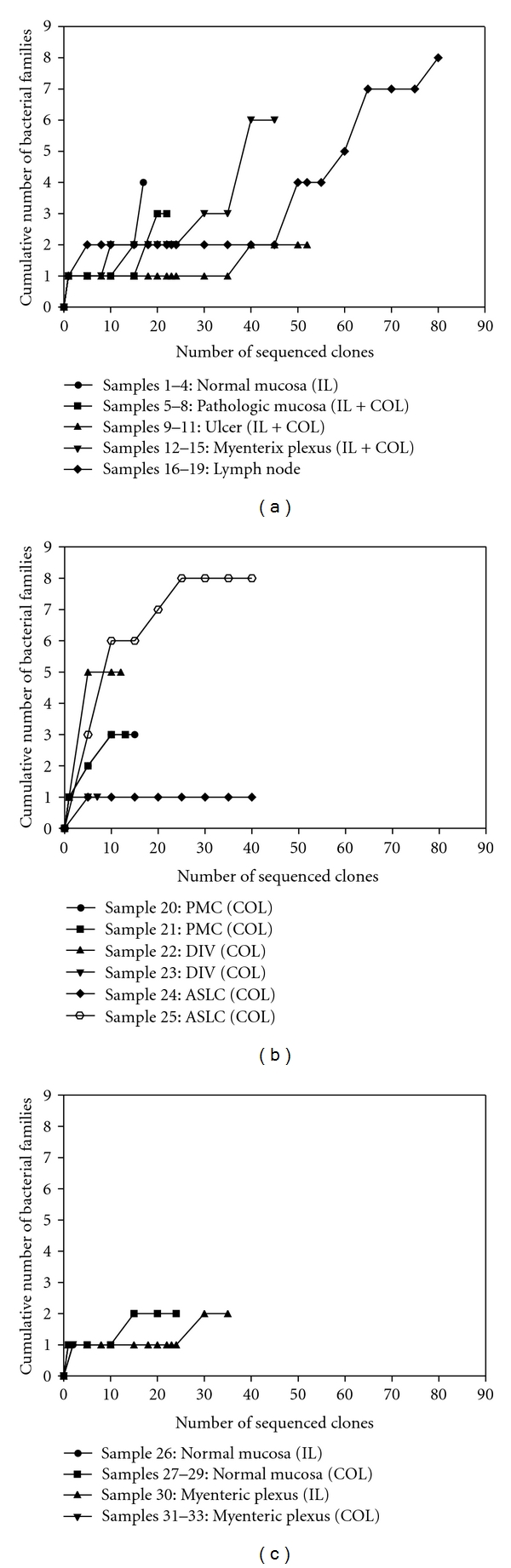
Plots of the cumulative numbers of detected genuinely present bacterial families versus the total number of bacterial sequences analysed for CD patients (a), disease controls (b), and healthy controls (c). ASLC: acute self-limited colitis, PMC: pseudomembranous colitis, DIV: diverticulitis, AC: adenocarcinoma. IL: ileum, COL: colon.

Amplified DNA with the predicted length of 1 kb was obtained after 2 PCR rounds in all 33 tissue samples from the 13 patients. The external positive control yielded the expected product and the negative controls remained blank after 30 cycles each. Sequencing of plasmid inserts after cloning resulted in 1161 sequences longer than 100 base pairs. Overall, 410 of these (35%) originated from genuinely present bacteria belonging to 4 phyla: Proteobacteria (51%), Firmicutes (28%), Actinobacteria (12%), and Bacteroidetes (9%). These 410 sequences could be further classified into 20 different families, and 147 sequences (36%) were identified unambiguously at the species level: *Escherichia coli* (17.5%), *Kocuria rosea* (6.6%), *Propionibacterium acnes* (2.9%), *Enterococcus faecalis* (2.2%), *Lactobacillus rhamnosus* (1.7%), *Streptococcus mitis* (1.7%), *Eubacterium rectale* (1.5%), *Bacteroides fragilis* (0.7%), *Clostridium nexile* (0.2%), *Bacteroides eggerthii* (0.2%), and *Bacteroides uniformis* (0.2%) (Table 5). No *Faecalibacterium prausnitzii* was identified on species level in the CD cases. Of the 751 remaining sequences, 55 were due to primer misalignment, 3 were likely chimeric, 187 originated from human DNA, and 506 were derived from probable contaminants belonging to the classes of the Alpha- and Betaproteobacteria and the families Pseudomonadaceae and Staphylococcaceae. Amplified contaminating DNA was significantly more commonly encountered in PCR products from microdissected versus non-microdissected tissues (71.0% and 1.4% of all bacterial sequences, resp.). The same applied to the proportion of sample-derived sequences of human instead of bacterial origin (47.0% and 2.4% in microdissected versus non-microdissected tissues, resp.) (Fisher's Exact test: *P* < 0.001 in both occasions). The cumulative numbers of detected genuinely present bacterial families plotted against the total numbers of analysed bacterial sequences revealed increasing slopes for most microdissected samples, while reaching asymptotic values for the non-microdissected tissues (Figure 2).

### 3.2. Comparison of the Microbiota in Crohn's Disease and Controls

Microdissected normal ileal mucosa from a healthy control yielded only 1 sequence, which was derived from the family of the Enterobacteriaceae. Normal colon mucosa yielded more sequences, pointing to a more abundant adherent microbiota which also contained strictly anaerobic bacteria (Bacteroidetes). Microbiota diversity was higher in the disease controls, with variation even between cases with an identical diagnosis. For example, one case of acute self-limited colitis showed a preponderance of sequences derived from Enterobacteriaceae while no potential pathogens were detected in the other patient's sample. Similarly, in 1 case of pseudomembranous colitis (PMC) all sequences were derived with certainty from *Lactobacillus rhamnosus*, while in the second case the microbiota was diverse but did not contain *Clostridium difficile*. One diverticulitis sample was completely colonized by Enterobacteriaceae, while the other showed a predominantly anaerobic adherent microbiota. Overall however, sequence analysis of the PCR products indicated that there might be a shift towards a nonstrictly anaerobic microbiota in disease controls versus healthy controls (Fisher's Exact test: *P* = 0.0243).

Histological normal microdissected ileal mucosa in Crohn's disease contained a fairly abundant, mixed aerobic—anaerobic microbiota. With ulceration, there was an increase in both the number of detectable bacterial sequences and the fraction of nonstrictly anaerobes (67% versus 62% in normal mucosa). This underrepresentation of anaerobes in ulcers was retained and seemed even more accentuated in so-called pathological mucosa, which shows macroscopic healing but is histologically still abnormal (Fisher's Exact test: *P* = 0.0138). An interesting feature of the myenteric plexus in Crohn's disease samples was the detection of DNA from a single bacterial species belonging to the family Legionellaceae. In this study, this biological signal was picked up exclusively in CD patients carrying at least 1 copy of either SNP8 or SNP12 of the *CARD15* gene (Table 1). No Legionellaceae were detected in controls or in the CD patient without CD-associated *CARD15* mutations. Tissue samples from ileocolonic lymph nodes in CD patients contained bacterial DNA, compatible with bacterial translocation. The translocating microbiota was mixed, with a higher proportion of strict anaerobes than in the bowel wall proper (39% versus 18%, Fisher's Exact test: *P* = 0.0007).

## 4. Discussion

The role of the gut microbiota in the etiopathogenesis of Crohn's disease is a hotly debated topic, and many experimental protocols have been devised to investigate this problem. In this study, we applied broad-range 16S rDNA PCR using universal primers, followed by sequencing of PCR products and bacterial identification based on the obtained sequences, an approach which has been used previously for faecal samples and mucosal biopsies [18]. The novelty of our approach is its application to microdissected normal tissue structures or CD-associated lesions such as ulcerations and architecturally abnormal, chronically inflamed mucosa. Theoretically, this combination would allow the identification of single bacterial species in well-defined locations, which may facilitate the detection of those microorganisms which are most likely involved in CD pathogenesis. To this end, results from CD cases were also compared with those obtained in microdissected and non-microdissected disease and healthy controls.

Laser capture microdissection was performed on fresh-frozen transmural bowel biopsies obtained from surgical samples. As part of the standard protocol, patients underwent gut lavage prior to surgery, and broad-spectrum preoperative antibiotics were administered to diminish the risk of sepsis. Also, the surgical specimens were flushed with tap water to remove residual bowel contents before transmural biopsies were obtained. These interventions will certainly lower the overall amount of mucosa-adherent bacteria, but will not necessarily eliminate them or significantly distort the original composition of the microbiota [23].

In this study, we microdissected 4 different bowel wall regions which were chosen based upon their expected informative value with regard to disease pathogenesis. The first area is the mucosa, which was selected since the highest number of bacteria is expected in this location [24]. Additionally, in CD a distinction can be made between structurally normal and pathological mucosa, which may be macroscopically intact but histologically abnormal. Ulcer beds are another lesion of interest, given their observable histological progression, frequency, and severity in this disease. The myenteric plexus is a well-recognizable histological landmark in bowel resections and may thus serve as a tissue sample suitable for the study of bacteria or bacterial products in passage through the bowel wall. Another important though not universal or entirely specific feature of CD is epithelioid granulomas. Two previous studies applying LCM and molecular bacteriological detection methods have demonstrated that these lesions can contain DNA from MAP and *Escherichia coli* [25, 26]. Another study from our group, using LCM and a universal bacterial PCR, suggested that the microbiota contained in granulomas may be even more diverse [27]. Based on these data, we now hypothesize that granulomas in Crohn's disease may act as areas of containment for all kinds of translocating bacteria. Testing this hypothesis would require a large-scale investigation of granuloma tissue obtained from surgical samples of patients with various genetic backgrounds, disease locations, and phenotypes. This is obviously beyond the scope of the current study. As an alternative, we chose to examine mesenteric lymph nodes, which also serve as a collector and filter for all the drained lymphatic fluid from both normal and pathological bowel segments.

Almost half of the sequences which we obtained in this study were derived from Alpha- and Betaproteobacteria, Pseudomonadaceae, and Staphylococcaceae. According to most previous descriptions, these taxa are distinctly uncommon in both the normal faecal and mucosa-associated bowel microbiota. We therefore assume that they are contaminants, which may have been present in the intact form or only as residual DNA either in the tap water used to flush the specimens on the surface of presterilized gloves and consumables or even in ultrapure water used for molecular tests [28].

We first investigated the normal mucosa-adherent microbiota in the ileum and colon of “healthy” controls, which we defined as tissue samples taken from macroscopically normal bowel at a large distance from the tumor in patients operated upon for colorectal adenocarcinoma. Since these tumors were nonstricturing and since preoperative gut lavage was possible in each patient, we assume that the presence of a tumor did not influence the composition of the mucosal microbiota in our samples. Results were as expected with regard to both the quantitative and qualitative aspect: the ileum was scarcely populated while colon mucosa contained a more abundant microbiota with a high fraction of strictly anaerobic bacteria.

Bacterial infections are characterized by microbial population dynamics changing over time, which may moreover be dependent on extraneous variables such as the administration of anti- and possibly probiotics. Therefore we investigated 3 types of disease controls, that is, cases of acute self-limited colitis (a pathological entity which in some cases has an infectious origin), pseudomembranous colitis, and diverticulitis (which has also been associated with changes in the composition of the faecal microbiota) [29–31]. Our results illustrate the disturbance of the normal mucosal population patterns in these conditions. We did not specifically identify *Clostridium difficile* in the cases of PMC. However, it should be noted that the standard toxin assays are performed on faecal samples, and that neither the classic assay nor the PCR for the *tcdB* toxin gene is entirely specific when compared with the gold standard of sensitive bacterial culture [32, 33]. It can thus be expected that in some toxin-positive cases, *C. difficile* cannot be isolated from stool samples even with the best available culture methods. A fortiori this would apply to mucosal biopsies which provide a much smaller amount of starting material. Moreover it is not known whether *C. difficile* in pseudomembranous colitis is mucosa-associated or dwells in the bowel lumen or in the pseudomembranes. It is indeed difficult to pin-point the presence of specific bacterial pathogens, for which the spatial and temporal distribution during the course of the infectious disease is a priori unknown. Our results may also have been influenced by the previous administration of various courses of antibiotics or possibly even probiotics (*Lactobacillus rhamnosus*) in some patients. Investigators of the microbiota in Crohn's disease should therefore always take into account a history of prior antibiotic therapy or administration of probiotics in their study subjects.

The only objective difference between histological normal ileal mucosa in Crohn's disease cases and the healthy control patient was the greater ease with which DNA from genuinely present bacteria was detected in the former. We can thus speculate that the absolute number of mucosa-adherent bacteria is larger in CD patients compared to controls, which fits with previous reports in the literature [34, 35]. We may further assume that the composition of the mucosal microbiota will show additional changes with the development of ulcerations. There are at least 2 possibilities: some bacterial variants may induce ulcers (e.g., the adherent-invasive subtype of *Escherichia coli*), or the presence of an ulcer slough and underlying granulation tissue may confer a selective growth advantage to particular bacterial taxa. In this study, no specific pathogen was detected in microdissected ulcer beds in Crohn's disease. There were however indications that these lesions may show localized bacterial overgrowth, with shifts in the composition of the microbiota possibly associated with an altered physicochemical milieu.

Previous studies on faecal samples have demonstrated an abnormally high biodiversity of the microbiota in CD patients in remission. Some harmful bacteria are increased (e.g., *Bacteroides fragilis, *an opportunistic pathogen) and the amount of protective, butyrate producing Firmicutes (e.g., *Faecalibacterium prausnitzii*) is often diminished in these patients [16, 24, 36, 37]. This dysbiosis is apparently independent of disease location, presence of anatomical abnormalities secondary to inflammation or scarring, treatment with sulphasalazine or corticosteroids and even surgery. Ileocolonoscopy in these patients often shows mucosal healing, with residual signs of inactive chronic inflammation on biopsy. Similar histological features can be seen in some macroscopically normal bowel segments in surgical specimens of patients operated upon for complicated Crohn's disease. We therefore investigated whether the mucosa-associated microbiota is also persistently altered in such areas of microscopically “pathological mucosa.” Our results show that this is indeed the case, with a significant increase in nonstrictly anaerobic taxa when compared with structurally normal mucosa.

It has been previously proposed that viable bacteria may be capable of crossing the intestinal mucosal barrier and of trafficking to extraintestinal sites such as the mesenteric lymph nodes, the systemic circulation, the liver and the spleen [38, 39]. This “bacterial translocation” may be a phenomenon that occurs in healthy individuals without deleterious consequences. Translocation of endotoxins from viable or dead bacteria in very small amounts probably constitutes a physiologically important boost to the reticulo-endothelial system, especially to the Kupffer cell in the liver. The baseline rate of translocation in human studies is 5–10% [39, 40]. Previous studies also indicate that Enterobacteriaceae translocate much more efficiently than other bacteria (especially obligate anaerobes) [39]. Bacterial translocation would be further promoted by 3 major conditions: intestinal bacterial overgrowth, deficiencies in host immune defenses, and increased mucosal permeability or major structural damage to the intestinal mucosal barrier [38]. Under these conditions, which may all be present in active Crohn's disease, one may expect a significant increase in the abundance of potentially pathogenic bacteria or bacterial products in the deeper layers of the bowel wall or in the surrounding lymph nodes. We tested this hypothesis for 2 anatomical compartments: the myenteric plexus as a surrogate for bacterial trafficking within the bowel wall and mesenteric lymph node tissue as the ideal locus to detect an even more advanced and potentially more dangerous form of bacterial translocation.

The main difference between the myenteric plexus samples of CD patients and healthy controls was the presence of a particular Legionella species in some of the former, more specifically only in our CD patients carrying at least one copy of either SNP8 or SNP12 of the *CARD15* gene. The relevant PCR product could not be identified at the species level, but the closest relative was *Legionella lytica*. Legionellaceae are gram-negative bacteria, which are able to reproduce at temperatures between 25°C and 43°C and survive in temperatures of up to 55–60°C. They are therefore ubiquitous in natural and artificial aquatic environments [41]. Since this sequence was only detected in CD patients and not in controls, the corresponding Legionella species may fit in the hypothesis of the persistent, still-unknown, pathogen. It could then be placed next to *Mycobacterium avium *subsp. *paratuberculosis* and adherent-invasive *Escherichia coli* which were also initially detected in very few CD patients [8–11]. The second reason why this finding may be relevant to the study of the etiopathogenesis of Crohn's disease is the known association of myenteric plexitis in the proximal margin of surgical specimens with early clinical and histological disease recurrence in the neoterminal ileum [42, 43].

Finally, our results on mesenteric lymph node tissues confirm the occurrence and the potential extent of bacterial translocation in Crohn's disease. The biological diversity of the translocating microbiota was large, with sequences from 8 different families being detected. *Escherichia coli* was the single most frequently detected species. Since the ratio of facultative over strict anaerobic bacteria was 1.47, facultative anaerobes may translocate more efficiently at larger distances.

## 5. Conclusion

In this study, we investigated alterations in the mucosa-associated and translocating bowel microbiota in Crohn's disease patients, disease controls, and healthy controls. We used a new approach consisting of laser capture microdissection of selected microscopic structures, followed by broad-range 16S rDNA PCR with universal primers. Our findings in healthy and disease controls are compatible with previous culture-based and molecular studies. They underline the significance of biopsy location and prior or concurrent antimicrobial therapy for a correct interpretation of the composition of the mucosa-associated bowel microbiota. Our results in Crohn's disease point to important spatiotemporal alterations of the gut microbiome in this condition. The composition and abundance of the mucosa-associated microbiota changes with the presence of mucosal defects and seems to remain abnormal upon macroscopic healing. There are also indications of increased bacterial translocation, possibly by uncommon bacterial species which may find a new ecological niche in the inflamed bowel wall. Further investigations using the combination of LCM and universal 16S rDNA PCR may lead to a better understanding of the role of the bowel microbiota in the etiopathogenesis of Crohn's disease.

## Figures and Tables

**Table 1:** Patient characteristics.

Patient	Sex	Age	Diagnosis	*CARD15-*SNP8	*CARD15-*SNP12	*CARD15-*SNP13	Procedure
1 (*)	♂	32	CD	WT	WT	WT	Right hemicolectomy
2 (*)	♀	36	CD	HE	WT	WT	Right hemicolectomy
3 (*)	♂	22	CD	HE	HE	WT	Right hemicolectomy
4 (*)	♂	18	CD	HO	WT	WT	Right hemicolectomy
5	♂	38	ASLC	ND	ND	ND	Colonoscopy
6 (**)	♂	70	ASLC	ND	ND	ND	Colonoscopy
7	♀	17	PMC	ND	ND	ND	Colonoscopy
8 (^§^)	♂	64	PMC	ND	ND	ND	Colonoscopy
9	♀	75	DIV	ND	ND	ND	Colonoscopy
10 (^§§^)	♂	48	DIV	ND	ND	ND	Colonoscopy
11 (*)	♀	65	AC	WT	WT	WT	Right hemicolectomy
12 (*)	♀	55	AC	WT	WT	WT	Total colectomy
13 (*)	♀	65	AC	HE	WT	WT	Total colectomy

ASLC: acute self-limited colitis, PMC: pseudomembranous colitis, DIV: diverticulitis, AC: adenocarcinoma.

WT: wild type, HE: heterozygous, HO: homozygous, ND: Not determined.

SNP8: Arg702Trp, SNP12: Gly908Arg, SNP13: Leu1007fsinsC.

(*) Treated with preoperative antibiotic coverage (cefazoline 2 g IV + metronidazole 1.5 g IV at induction).

(**) Treated with amoxicillin for 2 weeks prior to colonoscopy.

(^§^) Clostridium difficile toxin assay positive.

(^§§^) Treated with amoxicillin + clavulanic acid and levoflaxacin for 10 days prior to colonoscopy.

**Table 2:** Histological structures and lesions selected for LCM.

Patient	Normal mucosa (**)	Pathological mucosa (*) (**)	Ulcer base (*)	Myenteric plexus (*)
1	IL (sample 1)	Il + COL (sample 5)	Il + COL (sample 9)	Il + COL (sample 12)
2	IL (sample 2)	Il + COL (sample 6)	NA	Il + COL (sample 13)
3	IL (sample 3)	Il + COL (sample 7)	Il + COL (sample 10)	Il + COL (sample 14)
4	IL (sample 4)	Il + COL (sample 8)	Il + COL (sample 11)	Il + COL (sample 15)
11	IL (sample 26)	NA	NA	IL (sample 30)
11	COL (sample 27)	NA	NA	COL (sample 31)
12	COL (sample 28)	NA	NA	COL (sample 32)
13	COL (sample 29)	NA	NA	COL (sample 33)

IL: ileum; COL: colon; NA: Not applicable.

(*) IL + COL pooled per patient.

(**) The superficial half of the mucosa, the surface epithelium, and the adherent mucus were microdissected.

**Table 3:** Tissue samples which were not microdissected.

Patient	Lymph node	Mucosal biopsy
1	IL + COL (sample 16)	NA
2	IL + COL (sample 17)	NA
3	IL + COL (sample 18)	NA
4	IL + COL (sample 19)	NA
5	NA	COL (sample 20)
6	NA	COL (sample 21)
7	NA	COL (sample 22)
8	NA	COL (sample 23)
9	NA	COL (sample 24)
10	NA	COL (sample 25)

IL: ileum; COL: colon; NA: Not applicable.

**Table 4:** PCR primers.

Primer	Primer sequence (5′ → 3′)
342f	CTACGGGRSGCAGCAG
515f	GTGCCAGCMGCCGCGGTAATWC
1512r	TACGGYTACCTTGTTACGACTT

M = A : C, W = A : T, R = A : G, S = C : G, Y = C : T (all 1 : 1).

**Table 5:** Distribution of bacterial taxa over the 33 tissue samples (*) (**).

Identification	CD					Disease controls						Healthy controls			
	IL, normal mucosa (samples 1–4)	IL+COL, pathological mucosa (samples 5–8)	IL+COL, ulcer (samples 9–11)	IL+COL, myenteric plexus (samples 12–15)	lymph node (samples 16–19)	COL, ASLC (sample 20)	COL, ASLC (sample 21)	COL, PMC (sample 22)	COL, PMC (sample 23)	COL, DIV (sample 24)	COL, DIV (sample 25)	IL, normal mucosa (sample 26)	COL, normal mucosa (samples 17–29)	IL, myenteric plexus (sample 30)	COL, myenteric plexus (samples 31–33)
Total number of sequences (domain bacteria)	18	22	52	45	79	15	14	12	7	40	38	1	24	35	8
Phylum XII. Proteobacteria															
Class III. Gammaproteobacteria															
Order III. Xanthomonadales															
Family I. Xanthomonadaceae				11											
Order VI. Legionellales															
Family I. Legionellaceae				51											
Order IX. Pseudomonadales															
Family II. Moraxellaceae				18											
Order XII. Aeromonadales															
Family II. Succinivibrionaceae					1										
Order XIII. Enterobacteriales															
Family I. Enterobacteriaceae	56	68	67		15	53				5	32	100			
*Escherichia coli *					43					95					
Order XIV. Pasteurellales															
Family I. Pasteurellaceae					1						8				
Class V. Epsilonproteobacteria															
Order I. Campylobacterales															
Family I. Campylobacteraceae								8							
Phylum XIII. Firmicutes															
Class I. Clostridia															
Order I. Clostridiales															
Family I. Clostridiaceae	28				13			8			8				
*Clostridium nexile *					1										
Family II. Lachnospiraceae	11	5			5						18				
Family IV. Eubacteriaceae					1						8				
*Eubacterium rectale *											16				
Family VII. Acidaminococcaceae			33								3				
Class III. Bacilli															
Order I. Bacillales															
Family I. Bacillaceae							7								100
Order II. Lactobacillales															
Family I. Lactobacillaceae							7	8							
*Lactobacillus rhamnosus *									100						
Family IV. Enterococcaceae							21								
*Enterococcus faecalis *							64								
Family VI. Streptococcaceae				4				8			3		50		
*Streptococcus mitis *								58							
Phylum XIV. Actinobacteria															
Class I. Actinobacteria															
Order I. Actinomycetales															
Family I. Micrococcaceae														9	
*Kocuria rosea *				2										74	
Family I. Propionibacteriaceae				13										3	
*Propionibacterium acnes *	6	27												14	
Phylum XX. Bacteroidetes															
Class I. Bacteroidetes															
Order I. Bacteroidales															
Family I. Bacteroidaceae					14	7					3		50		
*Bacteroides fragilis *					4										
*Bacteroides eggerthii *								8							
*Bacteroides uniformis *											3				
Family III. Porphyromonadaceae					1										
Family IV. Prevotellaceae						40									

(*) Numbers indicated per taxon are percentages of the total number of sequences indicated in the first row.

(**) Empty cells indicate value of 0%.
